# Hybrid SSF/SHF Processing of SO_2_ Pretreated Wheat Straw—Tuning Co-fermentation by Yeast Inoculum Size and Hydrolysis Time

**DOI:** 10.1007/s12010-016-2229-y

**Published:** 2016-09-08

**Authors:** B. Cassells, K. Karhumaa, V. Sànchez i Nogué, G. Lidén

**Affiliations:** 1 Department of Chemical Engineering, Lund University, Box 124, 221 00 Lund, SE Sweden; 2 Novozymes A/S, Krogshoejvej 36, 2880 Bagsvaerd, SE Denmark; 3 C5 Ligno Technologies in Lund AB, P.O. Box 124, 221 00 Lund, SE Sweden; 4 Hansa Medical AB, P.O. Box 785, -220 07 Lund, SE Sweden; 5 National Bioenergy Center, National Renewable Energy Laboratory, 15013 Denver West Parkway, Golden, CO 80401 USA

**Keywords:** Bioethanol, Xylose fermentation, SSF, Yeast inoculum size, In situ detoxification

## Abstract

Wheat straw is one of the main agricultural residues of interest for bioethanol production. This work examines conversion of steam-pretreated wheat straw (using SO_2_ as a catalyst) in a hybrid process consisting of a short enzymatic prehydrolysis step and a subsequent simultaneous saccharification and fermentation (SSF) step with a xylose-fermenting strain of *Saccharomyces cerevisiae*. A successful process requires a balanced design of reaction time and temperature in the prehydrolysis step and yeast inoculum size and temperature in the SSF step. The pretreated material obtained after steam pretreatment at 210 °C for 5 min using 2.5 % SO_2_ (based on moisture content) showed a very good enzymatic digestibility at 45 °C but clearly lower at 30 °C. Furthermore, the pretreatment liquid was found to be rather inhibitory to the yeast, partly due to a furfural content of more than 3 g/L. The effect of varying the yeast inoculum size in this medium was assessed, and at a yeast inoculum size of 4 g/L, a complete conversion of glucose and a 90 % conversion of xylose were obtained within 50 h. An ethanol yield (based on the glucan and xylan in the pretreated material) of 0.39 g/g was achieved for a process with this yeast inoculum size in a hybrid process (10 % water-insoluble solid (WIS)) with 4 h prehydrolysis time and a total process time of 96 h. The obtained xylose conversion was 95 %. A longer prehydrolysis time or a lower yeast inoculum size resulted in incomplete xylose conversion.

## Introduction

Wheat straw is one of the main agricultural feedstocks for lignocellulosic ethanol production, and has—among others—been used in demonstration-scale facilities in Kalundborg, Denmark (Dong) [1], Salamanca, Spain (Abengoa) [2], and Straubing, Germany (Clariant) [3], and in the commercial-scale facility in Crescentino, Italy (Beta Renewables) [4]. Wheat straw contains around 35 % glucan and 20 % xylan together with minor amounts of arabinan (e.g., [5]), and the main challenge in fermentation of wheat straw hydrolysates is thus to accomplish an efficient conversion of both glucose and xylose. Furthermore, this needs to be done in the presence of various inhibitors in the hydrolysate, most of which are generated or liberated in the pretreatment process (e.g., [6], [7]). *Saccharomyces cerevisiae* (Baker’s yeast) is a proven robust organism in the traditional sucrose or starch-based ethanol industry and has been genetically engineered for efficient fermentation of lignocellulose-derived sugar mixtures (recently reviewed in [8]). A number of recombinant strains are now available, also commercially. This enables the use of engineered *S. cerevisiae* for conversion of xylose-rich hydrolysates. Fermentation inhibitors derived from lignocellulose pretreatment, in particular acid catalyzed pretreatments, include furans—primarily the 2-furaldehyde (furfural) and 5-hydroxy-2-furaldehyde (5-hydroximethylfurfural or HMF)—carboxylic acids—primarily acetic acid—and phenolic compounds [9]. The inhibitory mechanisms and effects of these compounds, in particular the furan compounds, are well elucidated in numerous studies, reviewed by, e.g., [10]. Of particular significance is the fact that furans—and a number of other aldehydes—can be converted by the yeast [11]. Under anaerobic conditions, this takes place mainly by reduction, facilitated by various dehydrogenases [12–15]. Once the reduction has been completed, the inhibition of the glycolytic flux is substantially reduced.

The uptake and conversion of xylose in recombinant xylose-fermenting yeasts are influenced by other sugars, primarily glucose. This is partly an effect of competition in the sugar uptake systems, since xylose is primarily transported into the yeast cell by hexose transporters, which have a higher affinity for glucose [16]. The expression levels of these transporters are furthermore affected by glucose concentration levels [17]. In some cases, xylose consumption is aided by simultaneous glucose consumption, compared with the rate of xylose consumption only. This effect may be even more pronounced in the presence of inhibitors. The relative concentration of glucose and xylose in the medium is strongly affected by the chosen process option. In a true separate hydrolysis and fermentation (SHF) process design, i.e., in a process where the enzymatic hydrolysis of the material is completely made before fermentation, the initial glucose to xylose concentration ratio from wheat straw will be in the order of 2:1—reflecting the carbohydrate composition of the raw material. In a simultaneous saccharification and fermentation process (SSF), i.e., in a process where the enzymatic hydrolysis takes place concomitant with the fermentation of released sugars, the ratio between the sugars will be rather different. Xylose as well as xylooligomers will be present in the liquid at the onset of an SSF as a result of the pretreatment, whereas glucose is both gradually released from glucan polymers and simultaneously consumed by the fermenting organism. By controlling enzyme dosage, temperature, and yeast concentration, the time profile of glucose and xylose concentrations may be tuned to facilitate co-consumption [18]. Process design is currently moving away from the base cases, i.e., SHF and SSF, toward hybrid processes. These may involve, as the case in the Crescentino plant, an initial high-temperature enzymatic hydrolysis, called prehydrolysis or viscosity reduction, followed by a lower-temperature SSF of the entire slurry [19]. In this way, a high rate of enzymatic hydrolysis—giving efficient liquefaction—is achieved initially, and by shifting to an SSF in which sugars are consumed, end-product inhibition of the cellulases is decreased.

The wheat straw structure is not highly rigid, which gives several possibilities with respect to pretreatment. A mild, autocatalytic, steam or hot water pretreatment using inherent acetyl groups is one option [20]. This will remove a substantial part of the hemicellulose from the fiber fraction, but results in a large fraction of oligomeric hemicellulose in the liquid fraction. These oligomers require hemicellulase components in the enzyme mixture used for hydrolysis. Alkaline pretreatment methods act by solubilizing lignin. Ammonia fiber expansion (AFEX) is often used on agricultural residues and gives effects both in terms of structural disruption and changed crystallinity of the material (see, e.g., [21]). A more recent development is extractive ammonia pretreatment, in which lignin is (partly) separated from the fiber in the ammonia stream after pretreatment [22]. The alkaline methods typically leave hemicellulose in the remaining fiber fraction. Alternatively, an acidic catalyst can be used in the steam pretreatment, in which case hemicellulose is more completely removed from the fiber fraction. Options include sulfuric acid, which is the most widely reported, but also weaker acids such as phosphoric acid or organic acids have been used [23]. The gaseous compound sulfur dioxide, SO_2_, is a further option. For a number of lignocellulosic raw materials, such as corn stover [24], aspen [25], sugarcane bagasse [26], and quinoa straw [27], this has resulted in a good overall yield of fermentable sugars, while minimizing the formation of degradation by-products. Conditions for high total recovery of both xylan—from the pretreatment—and glucose after enzymatic digestion of wheat straw using SO_2_ pretreatment have been described [28], but fermentation of such hydrolyzates has not yet been assessed.

The balanced design of a hybrid co-fermentation process for wheat straw hydrolysates requires the following: (a) a sufficient prehydrolysis time to allow enzymatic conversion of the solid fraction; (b) a suitable ratio of monosaccharides (glucose and xylose) to allow efficient xylose conversion during the fermentation; and (c) a sufficient yeast concentration in the broth to complete the conversion of the sugars. The feedstock composition and pretreatment have a strong influence on needed time for hydrolysis and the levels of inhibitors. The yeast concentration affects the volumetric consumption of sugars not only directly but also indirectly through the bioconversion of inhibitors. The volumetric rate will likely be higher when more yeast is inoculated, but not necessarily in a linear manner due to exposure time effects. Since there is a cost for producing yeast biomass, the yeast concentration should not be larger than necessary. In the present study, the design of hybrid saccharification and co-fermentation (SSCF) processes for SO_2_-pretreated wheat straw using an industrial xylose-fermenting strain of *S. cerevisiae* is outlined based on digestibility of the material and the fermentation performance in the pretreatment liquid at different yeast inoculum sizes.

## Materials and Methods

### Raw Materials

Wheat straw, locally harvested and dried in the field (Johan Håkansson Lantbruksprodukter, Lunnarp, Sweden), was milled and sieved into 1- to 10-mm pieces. The straw was soaked for 2 h in hot water at a solid to liquid ratio of 1:10 and subsequently pressed to 300 bars to reach a dry matter content of 50 %. The pressed straw was then steam-pretreated batch-wise, with an addition of 2.5 % SO_2_ (based on moisture content), at 210 °C for 5 min in a 10-L reactor [29]. After pretreatment, the material was stored at 4 °C until used. The composition of the pretreated wheat straw slurry (Table 1) was determined according to National Renewable Energy Laboratory (NREL) procedures [30, 31]. The water-insoluble solid (WIS) content of the material was determined to 12.1 % by washing repeatedly with deionized water over filter paper (Whatman No. 1) and drying over night at 105 °C.

**Table 1:** Composition of pretreated wheat slurry

Solid composition (% of WIS)		
	Average	Std dev
Glucan	55.6	1.9
Xylan	1.8	0.2
Galactan	n.d	–
Arabinan	n.d	–
Mannan	n.d	–
Lignin	35.7	0.1
Soluble components (g L^−1^)		
Sugars	Monomers	Total sugars (including monomers)
Glucose	1.6	6.2
Xylose	13.5	28.1
Galactose	n.d	n.d
Arabinose	0.9	2.0
Mannose	n.d	n.d
Inhibitors and degradation products		
Acetic acid	2.5	
HMF	0.3	
Furfural	3.5	

The solid composition is based on weight percentage of the WIS content, and the soluble components are reported in grams per liquid of liquid. The WIS content of the pretreated material was measured to 12.1 wt-%

*n.d.* not detected, i.e., below detection limit

### Enzymatic Hydrolysis

Enzymatic hydrolysis at low water-insoluble contents was performed in order to establish the maximum digestibility of the pretreated material. This was done by diluting the slurry with sterile water to 2 % WIS in capped bottles (100 mL final volume). The pH of the diluted slurry was then set to pH 5, and the bottles were placed in a temperature-controlled (45 °C) rotary shaker before adding an enzyme load of 0.1 g enzyme solution per gram of WIS (same as in SSF/SHF experiments). A hydrolysis experiment was also performed in a bioreactor at 30 °C in order to see how well the material was digested at the lower temperature.

### Cell Cultivation

The recombinant xylose-fermenting strain *S. cerevisiae* C5LT 1202 was used in all experiments. Strain C5LT 1202 is rationally engineered using the C5LT gene package technology [32] with the xylose reductase and xylitol dehydrogenase pathway in addition to auxiliary genes encoding xylose metabolism. Yeast cell mass was produced by an initial preculture in a shake flask, followed by aerobic cultivation on glucose, first in batch mode and finally in fed-batch mode.

The yeast was inoculated (from agar plate) in 300-mL shake flasks (liquid volume of 100 mL) containing 20 g L^−1^ glucose, 7.5 g L^−1^ (NH_4_)_2_SO_4_, 3.5 g L^−1^ KH_2_PO_4_, 0.74 g L^−1^ MgSO_2_∙7H_2_O, trace metals, and vitamins [33]. The cells were grown for 24 h at 30 °C and a starting pH of 5.5 in a rotary shaker at 180 rpm. Subsequently, the aerobic batch cultivation was performed in a 2.5-L bioreactor (Biostat A, B.Braun Biotech International, Melsungen, Germany) at 30 °C. The working volume was 0.7 L, and the medium contained 20.0 g L^−1^ glucose, 20.0 g L^−1^ (NH_4_)_2_SO_4_, 10.0 g L^−1^ KH_2_PO_4_, 2.0 g L^−1^ MgSO_4_, 27.0 mL L^−1^ trace metal solution, and 2.7 mL L^−1^ vitamin solution. The cultivation was initiated by adding 40.0 mL of the preculture to the bioreactor. The pH was maintained at 5.0 throughout the cultivation, by automatic addition of 3 M NaOH. Aeration was maintained at 1.2 L min^−1^, and the stirrer speed was kept at 800 rpm. When the glucose in the batch phase was depleted, as indicated by a sharp drop in carbon dioxide evolution rate (CER), the glucose feed was started and 1.0 L of feed (50 g/L glucose) was fed to the reactor. The feed rate was set initially to 0.04 L h^−1^ and increased linearly to 0.10 L h^−1^ during the 16-h fed-batch cultivation. The aeration during the fed-batch phase was maintained at 1.5 L min^−1^, and the stirrer speed was kept at 800 rpm.

After cultivation, the cells were harvested by centrifugation in 700-mL flasks using a HERMLE Z 513K centrifuge (HERMLE Labortechnik, Wehingen, Germany). The pellets were resuspended in 9 g L^−1^ NaCl solution to obtain a cell suspension with a cell mass concentration of 60 g dry weight per liter. The time between cell harvest and initiation of the following SSCF/shake flask fermentation was no longer than 3 h.

### Shake Flask Fermentations

One hundred-milliliter shake flask fermentation was performed in duplicates under anaerobic conditions in a 300-mL Erlenmeyer flask. The medium used for fermentation was the clarified liquid fraction obtained by separating the solids (with high-pressure filtration) from the pretreated hydrolyzate. Glucose (50 g/L) and xylose (30 g/L) were added to the pretreated hydrolysate liquid to mimic the expected concentrations reached after enzymatic hydrolysis of the fibers. The hydrolysate was supplemented with 0.5 g L^−1^ NH_4_H_2_PO_4_, 0.025 g L^−1^ MgSO_4_·7H_2_O, and 1.0 g L^−1^ yeast extract. During fermentation, the temperature was kept constant at 30 °C, while the pH was set initially to 5.3 and then left uncontrolled (pH did not drop below 5.0 in any fermentation). The precultivated yeast was added to start experiments at initial yeast concentrations of 1, 2, 4, and 8 g dry weight per liter. Liquid samples (2.5 mL) were withdrawn repeatedly during 48 h and analyzed for sugars and metabolites.

### Hybrid SSF/SHF Experiments

All experiments were carried out under anaerobic conditions using 2.5-L bioreactors (Biostat A, B.Braun Biotech International, Melsungen, Germany) with an initial WIS content of 10 % and a final working broth weight of 1.0 kg. The experiments were run for a total of 96 h. During the initial enzymatic hydrolysis phase, a temperature of 45 °C was maintained and when yeast was added (i.e., at the start of the SSF phase), the temperature was lowered and kept at 30 °C. The enzyme solution used was Cellic CTec 2 (Novozymes A/S, Bagsvaerd, Denmark) at a dose of 0.1 g enzyme solution per gram of WIS (corresponding to approximately 10 filter paper units (FPU)/g WIS). The pH was maintained at 5.0 throughout fermentation by automatic addition of 4 M NaOH. The wheat straw slurry was supplemented with 0.5 g L^−1^ NH_4_H_2_PO_4_, 0.025 g L^−1^ MgSO_4_·7H_2_O, and 1.0 g L^−1^ yeast extract at the start of the fermentation phase (same concentrations as for the shake flask fermentations). The initial yeast concentration was 2 and 4 g dry weight per liter. All experiments were carried out in duplicates.

## Results and Discussion

### Pretreatment and Enzymatic Digestibility

**Fig. 1:**
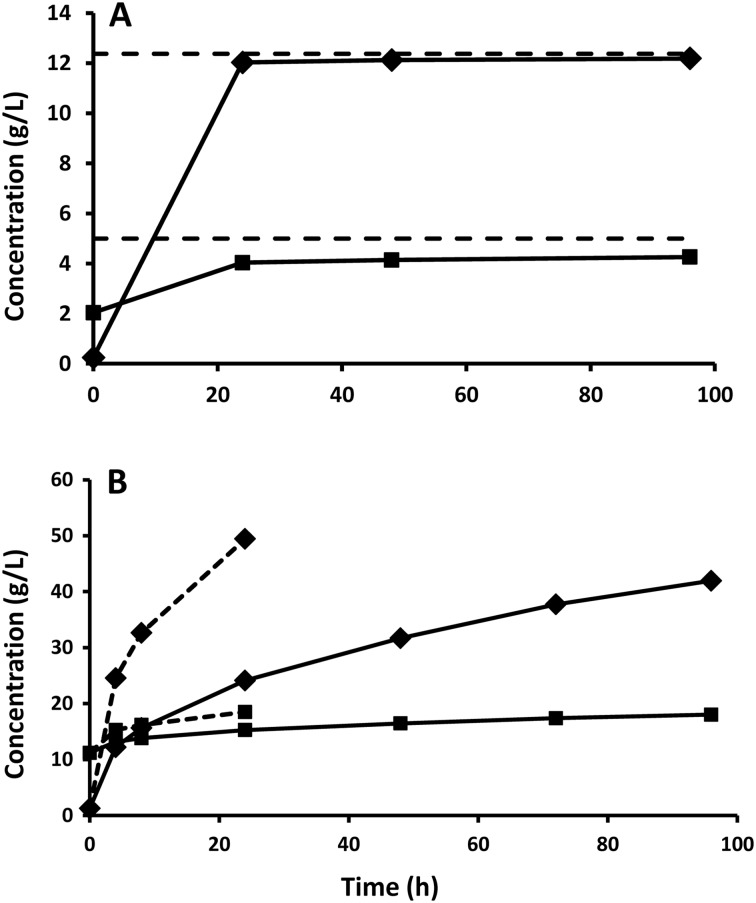
**a** Enzymatic hydrolysis at low (2 % WIS) solid loading and 45 °C. *Diamonds* represent glucose concentrations and *squares* represent xylose concentrations. The *dotted line* corresponds to the theoretical maximum for the respective sugars. **b** Enzymatic hydrolysis at 10 % WIS loading at 30 °C (*solid lines*) and 45 °C (*dotted lines*). *Diamonds* represent glucose concentrations and *squares* represent xylose concentrations

The pretreatment method used, with SO_2_ as catalyst, was expected to give a material with good digestibility, since the protocol used has previously resulted in high recovery of both xylose and glucose after enzymatic hydrolysis of materials such as sugar cane bagasse and quinoa stalks [26, 27]. The material analysis confirmed that the pretreatment resulted in an almost complete removal of the xylan fraction of the straw (Table 1). A large fraction of the xylan was recovered as oligomers rather than xylose monomers in the liquid. However, the chosen pretreatment conditions also generated a substantial amount of furfural (Table 1). The effect of the pretreatment on the digestibility of the material was assessed by enzymatic hydrolysis experiments at low WIS loading and high temperature (45 °C). An almost complete (>98 %) glucan degradation was achieved after only 24 h (Fig. 1a). A xylose yield as high as 85 % was achieved after 24 h confirming the efficiency of the chosen pretreatment protocol.

Enzymatic hydrolysis was also performed in bioreactors at a solid loading of 10 % WIS (Fig. 1b). The hydrolysis at 45 °C was not fully complete after 24 h (as the case for 2 % WIS), but the degree of hydrolysis reached was about 77 %. The optimal fermentation temperature for strain C5LT 1202 is 30 °C, thus SSF should be done at this temperature. At this lower temperature, the rate of hydrolysis was strongly decreased and the degree of conversion reached at 24 h was only 37 % (Fig. 1b).

### Fermentation

**Fig. 2:**
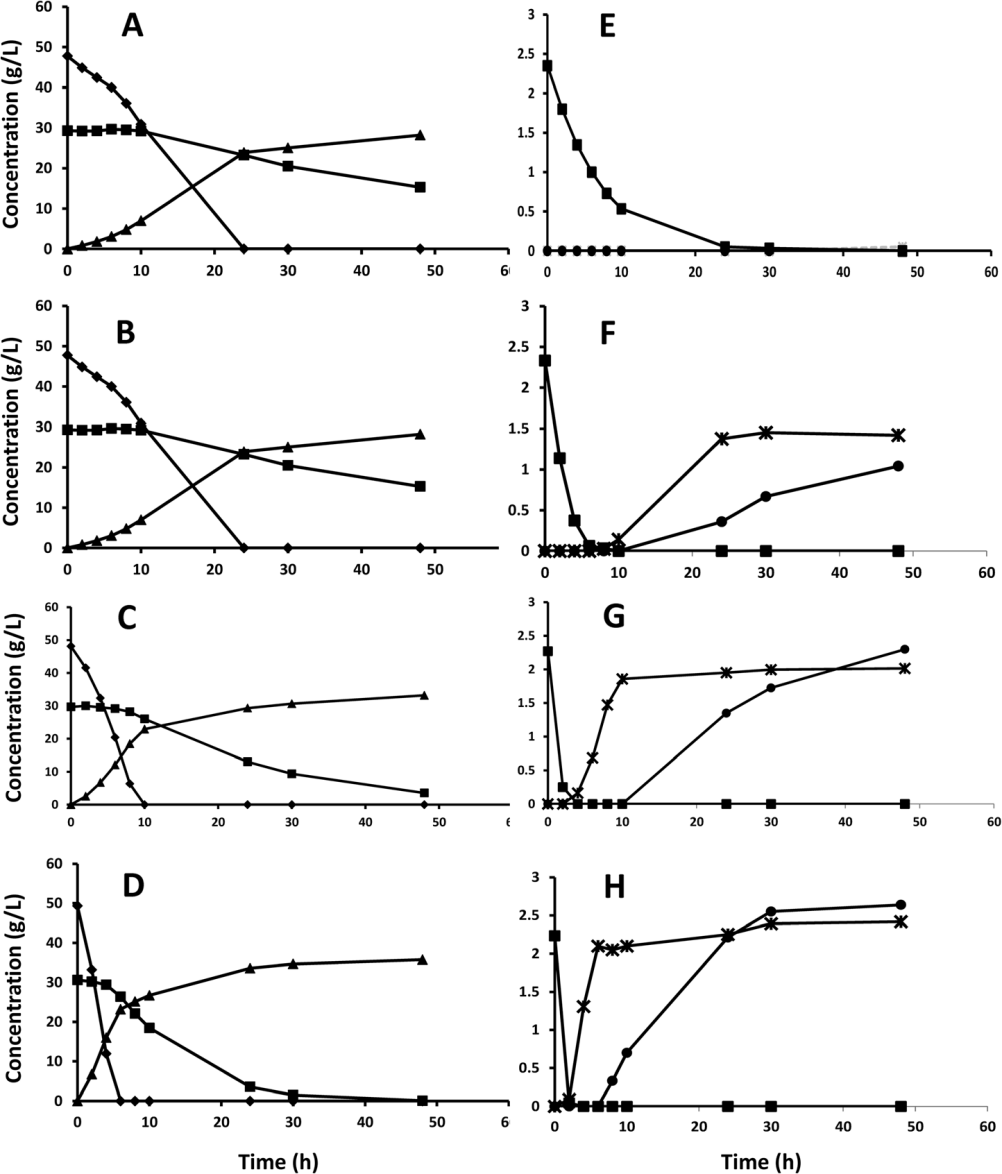
Shake flask fermentation of hydrolysis liquid with a yeast inoculum size of 1 g (**a**, **e**), 2 g (**b**, **f**), 4 g (**c**, **g**), and 8 g (**d**, **h**) dry weight per liter of yeast. *Diamonds* represent glucose, *squares* represent xylose, and *triangles* represent ethanol in **a**–**d**. *Squares* represent furfural, *stars* represent glycerol, and *circles* represent xylitol

**Fig. 3:**
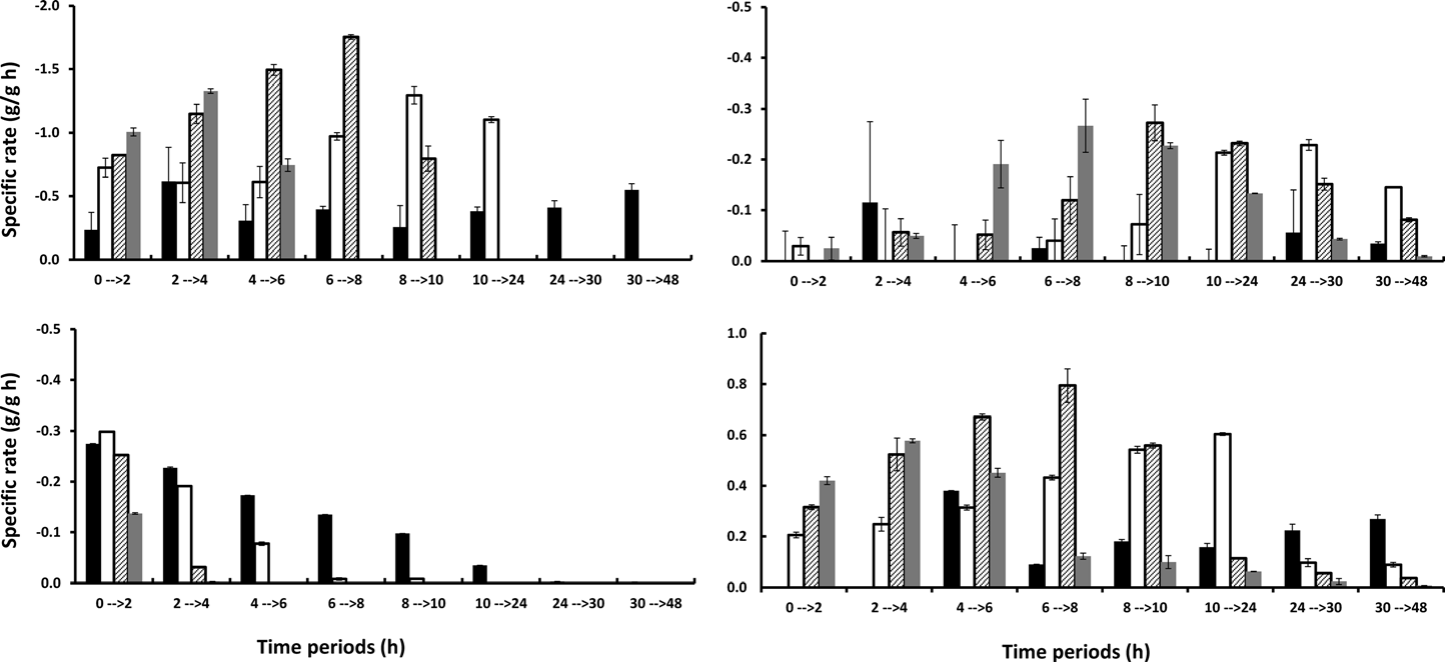
Specific uptake and production rates during the shake flask fermentation with different yeast inoculum sizes (Figs. 3 and 4). **a** Glucose uptake rate, **b** xylose uptake rate, **c** furfural uptake rate, and **d** ethanol production rate. The yeast inoculum size, going from 1 g/L up to 8 g/L, is indicated by the *bar patterns*; *black* is 1 g/L, *white* is 2 g/L, *hashed* is 4 g/L, and *gray* is 8 g/L. *The lower specific uptake rate of furfural at the highest yeast loading is an artifact of slow sampling, i.e., all furfural is depleted before the 2-h sample

The presence of furans in the liquid requires detoxification, which can be done in situ through the microbial reduction of furaldehydes to the corresponding alcohols [9]. The volumetric rate of detoxification will clearly depend on the yeast concentration, and the influence of the yeast inoculum size on in situ detoxification was for this reason investigated in shake flask fermentation of hydrolysis liquid. These experiments also test how in situ detoxification is affected by a prolonged exposure of the yeast to the harsh environment in the lignocellulose hydrolysate. A low yeast inoculum size gives a low volumetric rate of conversion, and thus, the yeast is exposed to the inhibitors, for example furfural, for a longer period of time. The fermentation results confirm that a higher yeast inoculum size gives higher volumetric sugar consumption and ethanol production rates (Fig. 2a–d) and that the rate of in situ detoxification is considerably faster (Fig. 2e–h). The rate of glucose consumption increases after the full conversion of furfural, and the rate of xylose consumption increases after glucose has been fully consumed. This is fully in line with reports on the lowering of sugar consumption in the presence of furfural [11, 34] and the competition for sugar transporters giving a sequential utilization [16]. The development of the specific uptake and production rates during the fermentation time courses (Fig. 3) indicate that rapid detoxification is of utmost importance for the fermentation outcome. Once furfural has been metabolized, similar specific sugar uptake rates regardless of yeast loading are obtained (Fig. 3a, b). There is therefore a time shift so that the “maximum rate” is reached earlier for the higher yeast inoculum size, since furfural is converted faster (Fig. 3c). The lowest yeast inoculum size (1 g/L) gives a qualitatively different time profile. Apparently, the cells do not recover completely within the studied time span even after furfural has been depleted. The specific conversion rate of furfural also seems to decrease the longer the cells are exposed to it (Fig. 3c). Both these observations are in line with previous reports on irreversible damage for high exposure [35] and underline the need for using a sufficiently high yeast inoculum size to avoid long exposure time to furfural and thereby decreased yeast performance.

**Fig. 4:**
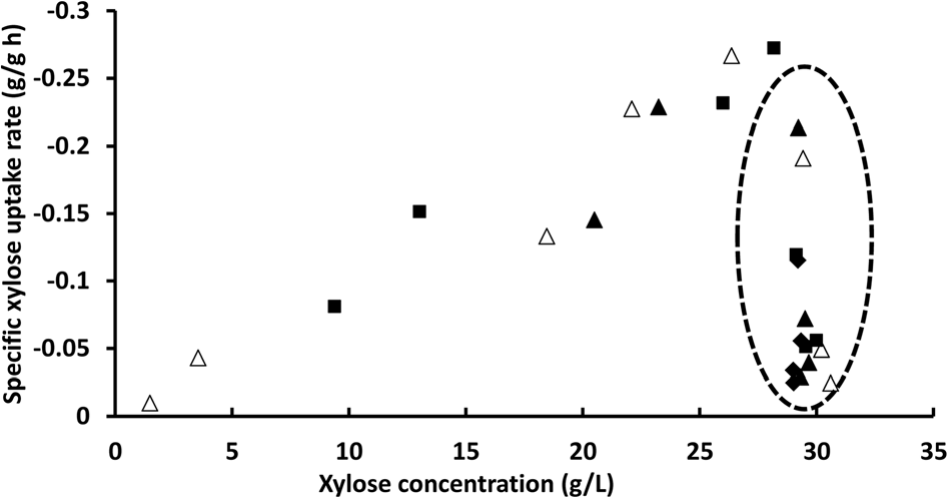
Specific xylose uptake rate shown as a function of the xylose concentration in the media throughout the time course of shake flask fermentation (Fig. 2). The *different symbols* indicate different yeast inoculum sizes (*diamonds* 1 g/L, *filled triangles* 2 g/L, *squares* 4 g/L, and *unfilled triangles* 8 g/L). The *dashed circle* indicates time-points where glucose was still present in the media, i.e., early samples before all glucose was consumed

In terms of xylose uptake, it appears that the specific xylose uptake for the examined strain is an almost linear function of the xylose concentration in the broth once the glucose is consumed (Fig. 4). This is in line with the known low affinity uptake kinetics for xylose, giving a concentration dependence on the uptake rate. In the presence of measureable glucose concentrations, the xylose uptake is furthermore strongly reduced, in agreement with competitive inhibition between the sugars.

### Hybrid SSF

**Fig. 5:**
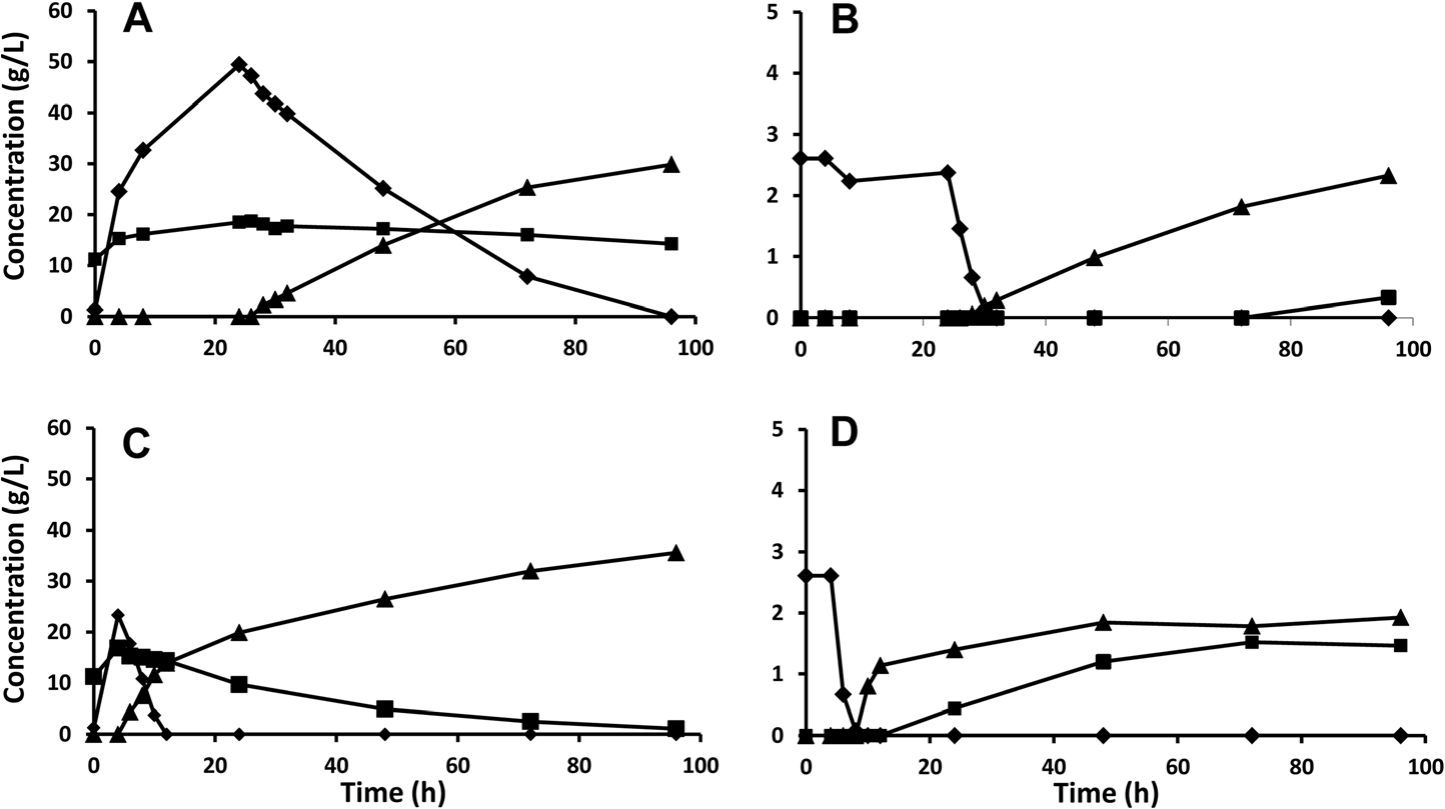
Concentration profiles for hybrid SSF/SHF experiments. In **a**, **b,** a 24-h prehydrolysis (45 °C) was performed before lowering the temperature to 30 °C and adding 2 g/L of yeast to start the fermentation. In **c**, **d**, a 4-h prehydrolysis (45 °C) was performed before lowering the temperature to 30 °C and adding 4 g/L of yeast to start the fermentation. Symbols used in **a**, **c**: *Diamonds* represent glucose, *squares* represent xylose, and *triangles* represent ethanol. Symbols used in **b**, **d**: *Diamonds* represent furfural, *squares* represent xylitol, and *triangles* represent glycerol

Finding the optimal yeast inoculum size to use in a SSF/SHF process will require a thorough techno-economical evaluation since the yeast loading both comes as a cost (production cost and overall yield loss due to increased sugar utilization) and as a potential saving due to increased productivity. It is also clear that the design will be a compromise between the efficient high-temperature hydrolysis and the lower temperature tolerated during fermentation. In this study, a simple design was chosen aiming for complete conversion of glucose and xylose in 96 h. From the fermentation experiments (described above), one can estimate that the specific glucose consumption rate is in the order 1.5 g/g h, the specific xylose consumption rate is in the order 0.2 g/g h, and the specific furfural conversion rate is in the order of 0.25 g/g h. In a hybrid SSF process with 24 h enzymatic hydrolysis (EH) at 45 °C, there is about 50 g/L glucose and 20 g/L xylose, and the furfural concentration is about 2.5 g/L. As a first simplification is it assumed that the glucose consumption rate is very low until all furfural has been converted and that no xylose consumption occurs until glucose concentration is zero. With these numbers, a yeast inoculum size of 2 g/L should give conversion of the furfural within about 5 h and subsequently conversion of all glucose within another 17 h. This would leave 48 h for conversion of xylose, which if taking place at maximum rate would require about 50 h. However, the experimental results proved different, and the glucose concentration was in fact not exhausted until at between 72 and 96 h (Fig. 5a). The furfural conversion rate agreed rather well with the estimate. The glucose concentration is somewhat more complicated to calculate, since glucose continues to be released during the SSF. Based on the ethanol production and using the same ethanol yield on consumed glucose (0.44 g/g) as in the separate fermentation experiments at 1 g/L (Fig. 2a), the glucose uptake rate can be estimated to a mere 0.6 g/g h, i.e., clearly lower than assumed. Consequently, the xylose consumption was also limited (43 %) and the technical ethanol yield obtained (based on glucose and xylose sugars in the pretreated material) was 0.32 g/g.

To ensure a complete xylose consumption, the time for EH should be decreased and/or the yeast inoculum size should be increased. In order to ensure that the specific glucose consumption would not fall due to decreased performance following furfural conversion, the yeast inoculum size was doubled. The initial free glucose was also reduced by 50 % from shortening the high-temperature EH to only 4 h (Fig. 5b). In this case, the hybrid SSF resulted in 95 % conversion of xylose, and the glucose concentration at the end was also negligible. A final ethanol concentration of 35 g/L corresponding to a technical yield of 0.39 g/g was reached, 17 % higher than the previous case. Further fine tuning may give a somewhat increased yield with a slightly lower yeast inoculum size.

## Conclusion

The present work illustrates the importance of yeast inoculum size for detoxification of pretreated wheat straw and how this needs to be taken into account when designing hybrid SSCF processes. Depending on the yeast strain used, one can anticipate at least three more or less sequential processes: in situ detoxification, glucose conversion, and finally xylose conversion. Increasing the initial time for high-temperature enzymatic hydrolysis in a hybrid process will speed up the hydrolysis of glucan in the fiber. However, it will also increase the concentration ratio between glucose and xylose in the liquid, which in turn will delay the onset of xylose fermentation. Furaldehyde inhibitors present in the medium will delay the start of glucose fermentation, and a sufficient yeast inoculum must be chosen with this in mind.
